# Effects of Obesity Reduction on Physical Function, Inflammation and Osteoarthritis in Older Adults

**DOI:** 10.1093/geroni/igab046.3692

**Published:** 2021-12-17

**Authors:** Michael Borack, Abigail Holt, Kathryn Porter Starr, Jamie Rincker, James Coppock, Connie Bales, Lou DeFrate, Amy McNulty

**Affiliations:** 1 Duke University School of Medicine, Durham, North Carolina, United States; 2 Duke University, Durham, North Carolina, United States; 3 Duke University School of Medicine, Hillsborough, North Carolina, United States

## Abstract

Age-related increases in chronic inflammation lead to reduced physical function via damage to muscle and joints and contribute to osteoarthritis (OA) risk. Obesity in older adults with OA further exacerbates inflammatory damage. Whether obesity reduction can lessen inflammation and improve OA is unknown; however, novel biomarkers may provide an answer. We completed a 6-mo. weight loss intervention (-500 kcal/day), studying blood biomarkers of inflammation and cartilage damage along with physical function in obese older adults with (OA+; n=39) and without an OA diagnosis (OA-; n=20). Participants were aged > 60 yrs (mean = 70.2±6.0) and obese (BMI =34.6±4.7 kg/m2). At endpoint, weight loss was -6.3±4.0% and -5.8±4.1% in OA+ and OA-, respectively, with no group difference. Change scores for function for OA+ and OA- were: Short Physical Performance Battery score (+1.7±1.3 and +2.1±1.5), 8 ft up and go (-0.7±1.0 and -0.9±1.12 sec) and 6 min walk (+31.4±105.1 and +39.5±57.4 meters). All improved from baseline (p<0.05), with no group difference. Concerning blood biomarkers, there was a decrease (p<0.05) in cartilage oligomeric matrix protein (COMP: OA biomarker), indicating a potential benefit for OA. Change in COMP also differed between groups; OA- had a greater (p<0.05) reduction than OA+. Pooled results showed improved adiponectin (p<0.05), with no group difference. There were no changes for CRP, CTX-1, IL-6 and TNF-α. Our novel findings link early intervention with better reduction of OA risk and inflammation in obese older adults and also show important benefits for improved physical function regardless of OA status.

